# Seroepidemiology of dengue virus infection among adults during the ending phase of a severe dengue epidemic in southern Taiwan, 2015

**DOI:** 10.1186/s12879-019-3946-y

**Published:** 2019-04-24

**Authors:** Yu-Wen Chien, Hsiang-Min Huang, Tzu-Chuan Ho, Fan-Chen Tseng, Nai-Ying Ko, Wen-Chien Ko, Guey Chuen Perng

**Affiliations:** 1 Department of Public Health, College of Medicine, National Cheng Kung University, Tainan, Taiwan; 2 Department of Occupational and Environmental Medicine, National Cheng Kung University Hospital, College of Medicine, National Cheng Kung University, Tainan, Taiwan; 3 Institute of Basic Medical Sciences, College of Medicine, National Cheng Kung University, Tainan, Taiwan; 4 Department of Nursing, National Taipei University of Nursing and Health Sciences, Taipei, Taiwan; 5 National Institute of Infectious Diseases and Vaccinology, National Health Research Institutes, Tainan, Taiwan; 6 Department of Nursing, College of Medicine, National Cheng Kung University, Tainan, Taiwan; 7 Department of Medicine, College of Medicine, National Cheng Kung University, Tainan, Taiwan; 8 Department of Microbiology and Immunology, College of Medicine, National Cheng Kung University, No. 1, University Road, Tainan, 70101 Taiwan; 9 Center of Infectious Disease and Signaling Research, National Cheng Kung University, Tainan, Taiwan

**Keywords:** Adult dengue, Seroprevalence, Dengue vaccine, Asymptomatic, Flaviviruses

## Abstract

**Background:**

A severe dengue epidemic occurred in 2015 which resulted in over 22,000 laboratory-confirmed cases. A cross-sectional seroprevalence study was conducted during the ending phase of this epidemic to evaluate the true incidence of dengue virus (DENV) infection and the level of herd immunity.

**Methods:**

Adult residents in three administrative districts with high dengue incidence were recruited; workers in two districts with intermediate dengue incidence were also recruited for comparison. DENV-specific IgM and IgG were tested using commercial enzyme-linked immunosorbent assays. DENV RNA was detected using commercial quantitative real-time reverse transcriptase polymerase chain reaction assay. Univariate and multivariate logistic regressions were performed to identify risk factors for recent and past DENV infection.

**Results:**

The overall seroprevalence of anti-DENV IgM and IgG in 1391 participants was 6.8 and 17.4%, respectively. The risk of recent DENV infection increased with age, with the elderly having the highest risk of infection. Living in areas with high incidence of reported dengue cases and having family members being diagnosed with dengue in 2015 were also independent risk factors for recent DENV infection. One sample was found to have asymptomatic viremia with viral load as high as 10^5^ PFU/ml.

**Conclusions:**

Comparing the seroprevalence of anti-DENV IgM with the incidence of reported dengue cases in 2015, we estimated that 1 out of 3.7 dengue infections were reported to the surveillance system; widespread use of rapid diagnostic tests might contribute to this high reporting rate. The results also indicate that the overall herd immunity remains low and the current approved Dengvaxia® is not quite suitable for vaccination in Taiwan.

## Background

Dengue fever, an important mosquito-borne infectious disease globally, is caused by four distinct dengue virus serotypes (DENV1–4) which belong to the genus *Flavivirus*, family *Flaviviridae*. Infection by DENV can result in inapparent infection, mild flu-like illness, classical dengue fever (DF), or severe dengue [1]. Over the past 50 years, the incidence of dengue has risen 30-fold worldwide with continuing geographic expansion to new regions, probably as a consequence of global climate change, increasing frequency of travel, and unplanned urban development [2, 3].

Taiwan is located in East Asia with the Tropic of Cancer passing through near centrally. *Aedes aegypti* is only prevalent in tropical southern Taiwan while *Aedes albopictus* can be found on the whole island [4]. Historically, several dengue epidemics had been documented in the first half of twentieth century and the most severe one occurred in 1942–43 when an estimated five-sixths of the population on the island were infected, presumably due to tremendous human migration during World War II [5–7]. Since then, no dengue outbreaks had been reported on the main island of Taiwan until 1987, probably because international travel was restricted under the martial law between 1949 and 1987 [6, 8, 9]. After 1987, dengue epidemics with various sizes have been found in southern Taiwan almost every year, mainly in Kaohsiung City [5, 6]. However, dengue is currently not considered to be endemic in Taiwan [8]; outbreaks of dengue are presumed to be caused by imported DENV from neighboring Southeast Asian countries.

Tainan City covers an area of 2191.63 km^2^ in southern Taiwan and has a population of approximately 1.88 million. Tainan usually had small dengue outbreaks involving only dozens or hundreds of cases every year before 2015, except in 2007 when more than 1800 confirmed cases were notified in a DENV1 epidemic. However, a severe DENV2 epidemic occurred in 2015 which resulted in 22,777 laboratory-confirmed reported cases, of which 0.8% were fatal [10]; approximately 90% of the confirmed cases were adults and the elderly [10]. Reporting of dengue cases is required by law in Taiwan. However, the iceberg effect for DENV infection is well-known; those who are reported to the surveillance system only represent a small proportion of total infections, while numerous infections are unrecognized due to both asymptomatic infections as well as symptomatic cases not being reported. The aim of this study was to investigate the seroprevalence of DENV infection among adults during the end of the 2015 dengue epidemic in Tainan to estimate the true infection incidence and to evaluate the level of herd immunity.

## Methods

### Participants

**Fig. 1:**
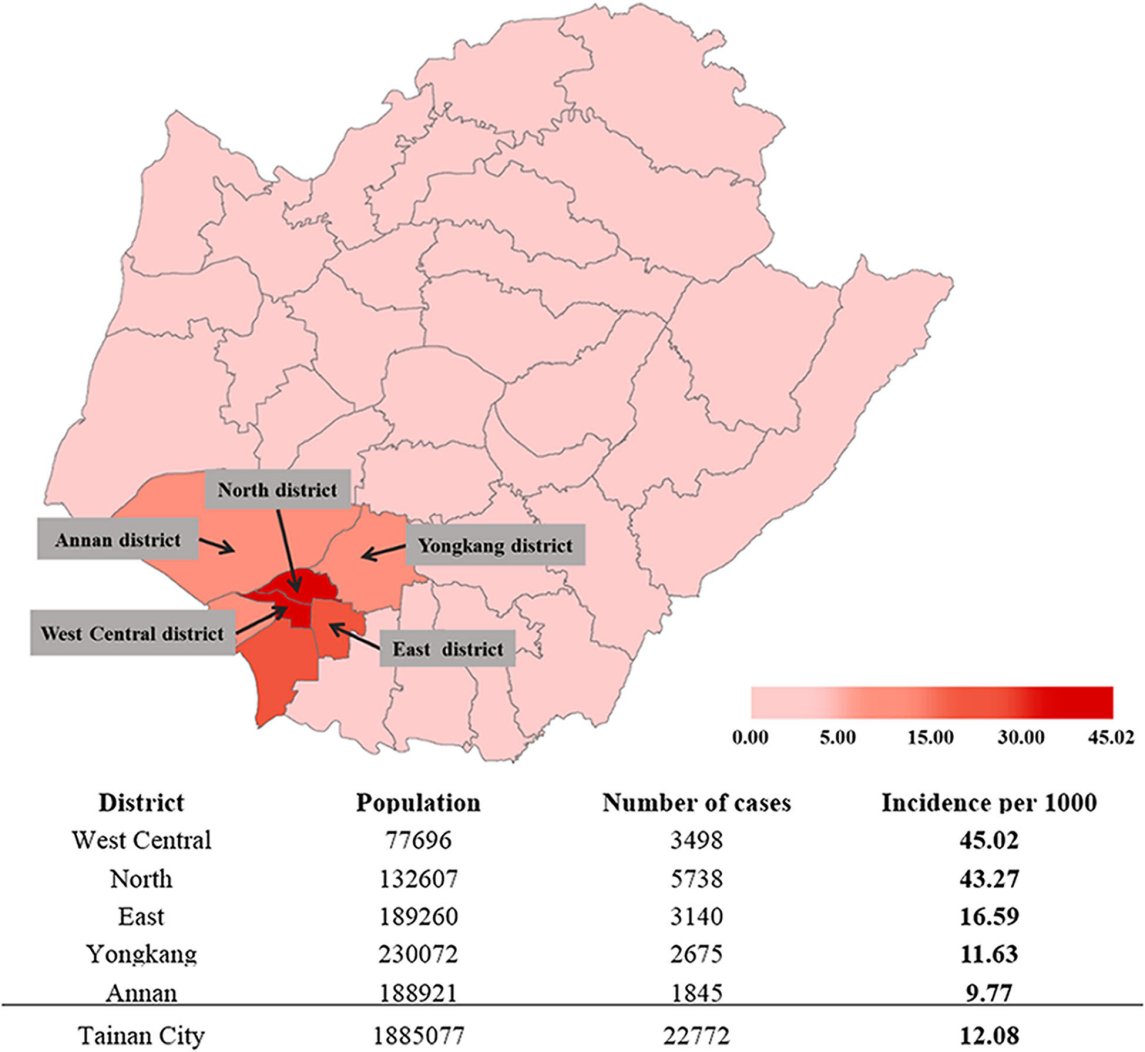
Incidence of reported dengue cases by administrative district in the 2015 dengue epidemic in Tainan, Taiwan and the distribution of five study sites in Tainan and their incidence [12]. The figure was created using ArcGIS version 10.5 software with base layers obtained from Government Open Data Platform in Taiwan (https://data.gov.tw/dataset/7441 and https://data.gov.tw/dataset/7442)

The 2015 dengue epidemic in Tainan began in May and remained at low activity in June and July; the number of confirmed cases dramatically increased in August and peaked in September, then rapidly declined in early October [11]. A cross-sectional seroepidemiologic survey was conducted between mid-October and late November in 2015. Due to the unexpectedness of this severe epidemic and limited resources at that time, a convenience sample of adult volunteers were recruited from residents in three administrative districts with high dengue incidence in Tainan: West Central District, North District and East District (Fig. 1). Recruitment stands were set up at local activity centers or near traditional markets to recruit volunteers. Workers who received annual health examinations in two factories located in two districts with intermediate dengue incidence (Annan District and Yongkang District) were also recruited for comparison; both of the factories had more than 1000 workers, manufacturing electronic parts and components. People aged ≥20 years were eligible for this study. Basic demographic information including age and gender, personal and family history of DENV infection were collected using a short questionnaire.

### Diagnostic assays

Blood samples were taken and kept on ice during transport and processing, and then stored at − 80 °C before serological testing. DENV-specific antibodies in sera were tested using a commercial anti-DENV IgM capture enzyme-linked immunosorbent assay (ELISA) (Standard Diagnostic, Kyonggi-do, South Korea) and an indirect anti-DENV IgG ELISA (Focus Diagnostics, Cypress, CA, USA) according to the manufacturers’ instructions. DENV RNA extracted from groups of 10 samples in a pool was detected by a validated commercial quantitative real-time reverse transcriptase polymerase chain reaction assay (qRT-PCR) [13]. Only samples from positive pools received individual qRT-PCR testing for cost consideration. The detailed methods for RNA extraction and qRT-PCR were performed as previously described [13].

### Statistical analysis

Seroprevalence of positive IgG and IgM against DENV were calculated, stratified by study site and age group. Age-standardized seroprevalence of anti-DENV IgG and IgM was also calculated for each study site using the age structure of the total population in Tainan City as the reference population. Univariate logistic regressions were performed to identify risk factors for recent and past DENV infection, and multivariate logistic regressions were also used to adjust for covariates. Results were considered statistically significant at the *p* < 0.05 level. These analyses were performed using SAS 9.4 (SAS Institute, Cary, NC, USA).

## Results

### Demographic characteristics of participants

The demographic characteristics of the participants were shown in Table 1. A total of 1391 adults were recruited in this study and the mean age of the study population was 44.6 years (range 20–88 years, standard deviation [SD] 16.4 years). There were more females (57.9%) than males (42.1%) in the study. Fifty-two (3.7%) of the study population reported that had been diagnosed with dengue in the past; 38 (2.7%) of them reported being diagnosed with dengue in 2015.

**Table 1:** Demographic distribution and characteristics of the study population

Characteristics	Number	Percent
Male		
Male	586	42.1
Female	805	57.9
Age group		
20–34	510	36.7
35–49	344	24.7
50–64	330	23.7
≥ 65	207	14.9
Place		
West Central District	226	16.3
North District	175	12.6
East District	363	26.1
A factory in Annan District	325	23.4
A factory in Yongkang District	302	21.7
Diagnosed with dengue in 2015		
Yes	38	2.7
No	1353	97.3
Family diagnosed with dengue in 2015		
Yes	102	7.3
No	1289	92.7
Diagnosed with dengue at any time		
Yes	52	3.7
No	1339	96.3
Family diagnosed with dengue at any time		
Yes	121	8.7
No	1270	91.3

### Characteristics of participants with recent DENV infection

Ninety-five (6.8%) of the participants had detectable anti-DENV IgM. Table 2 showed the seroprevalence of anti-DENV IgM stratified by age and area of the recruited participants. In all, the seroprevalence increased with age and 15.9% of the elderly had recent DENV infection (Tables 2 and 3). Participants in West Central District had the highest seroprevalence of anti-DENV IgM (22.6%), followed by North District (7.4%) and East District (6.6%), and the seroprevalences in participants recruited from the two factories were much lower (Table 2). The age-adjusted seroprevalence also showed similar results; however, only a very small number of people aged ≥50 years were recruited in the two factories and thus the seroprevalences were not directly comparable even after age standardization (Table 2). Multivariate logistic regression showed that people aged 50–64 and ≥ 65 years had a significantly higher risk of recent DENV infection as compared to those aged 20–34 years (adjusted OR = 3.40 and 5.16, *p* = 0.0108 and 0.0009, respectively) (Table 3). Subjects inhabiting in West Central District were also an independent risk factor for recent DENV infection (Table 3). Furthermore, people who had family members living in the same house being diagnosed with dengue in 2015 also had a significantly higher risk of being infected in 2015 (adjusted OR 3.2, 95% CI 1.8–5.7) (Table 3). Finally, it was found that of the 38 participants who reported being diagnosed with dengue in 2015, 35 (92.1%) had detectable anti-DENV IgM.

**Table 2:** Seroprevalence of anti-dengue virus IgM stratified by age groups and study sites

	West Central	North District	East District	A factory in Ana District	A factory in Yongkang District	Total
Age group	+/N	+/N	+/N	+/N	+/N	+/N
	(%)	(%)	(%)	(%)	(%)	(%)
20–34	2/27	0/23	0/40	5/243	1/177	8/510
	(7.4)	(0.0)	(0.0)	(2.1)	(0.6)	(1.6)
35–49	9/48	2/40	1/65	0/77	1/114	13/344
	(18.8)	(5.0)	(1.5)	(0.0)	(0.9)	(3.8)
50–64	23/101	10/79	8/134	0/5	0/11	41/330
	(22.8)	(12.7)	(6.0)	(0.0)	(0.0)	(12.4)
≥65	17/50	1/33	15/124			33/207
	(34.0)	(3.0)	(12.1)			(15.9)
Total	51/226	13/175	24/363	5/325	2/302	95/1391
	(22.6)	(7.4)	(6.6)	(1.5)	(0.7)	(6.8)
Age-standardized	18.6%	5.4%	3.7%	0.6%	0.4%	7.1%

**Table 3:** Analysis of recent dengue virus infection among study population by selected characteristics

		Univariate analysis				Multivariate analysis		
Characteristics	No. positive/No. tested	(%)	Odds ratio	95% CI	*P* value	Adjusted OR	95% CI	*P* value
Gender								
Male	29/586	(5.0)	1.00	Referent	-	1.00	Referent	-
Female	66/805	(8.2)	1.72	(1.09,2.69)	0.0188	1.21	(0.74,1.99)	0.4463
Age group (y)					<.0001			0.0012
20–34	8/510	(1.6)	1.00	Referent	-	1.00	Referent	-
35–49	13/344	(3.8)	2.46	(1.01,6.01)	0.0474	1.49	(0.56,3.95)	0.4209
50–64	41/330	(12.4)	8.90	(4.12,19.25)	<.0001	3.40	(1.33,8.72)	0.0108
≥65	33/207	(15.9)	11.90	(5.39,26.26)	<.0001	5.16	(1.95,13.64)	0.0009
Place					<.0001			<.0001
West Central District	51/226	(22.6)	18.65	(7.31,47.59)	<.0001	5.92	(1.94,18.06)	0.0018
North District	13/175	(7.4)	5.14	(1.80,14.66)	0.0022	1.81	(0.53,6.11)	0.3416
East District	24/363	(6.6)	4.53	(0.08,2.22)	0.0024	1.53	(0.47,4.93)	0.4772
Factory in Yongkang	2/302	(0.7)	0.43	(1.71,12.02)	0.3109	0.40	(0.08,2.13)	0.2844
Factory in Annan	5/325	(1.5)	1.00	Referent	-	1.00	Referent	-
Family diagnosed with dengue in 2015								
No	71/1289	(5.5)	1.00	Referent	-	1.00	Referent	-
Yes	24/102	(23.5)	5.28	(3.15,8.85)	<.0001	3.20	(1.79,5.71)	<.0001

### Seroprevalence of anti-DENV IgG

Two hundred forty-two specimens (17.4%) were seropositive for anti-DENV IgG (Table 4). The percentage of participants with positive anti-DENV IgG also increased with age (Table 4 and Table 5). Similar to the results of anti-DENV IgM, individuals in West Central District had the highest seroprevalence of anti-DENV IgG; participants in North District and East District had similar seroprevalence overall, but the seroprevalence of anti-DENV IgG in North District was higher after age-standardization (Table 4). Multivariate logistic regression showed that age ≥ 50, living in West Central District and North District, and having family members being diagnosed with dengue in the past were independent risk factors for past DENV infection (Table 5). Anti-DENV IgG was detected in 36 (94.7%) of 38 participants reporting being diagnosed with dengue in 2015.

**Table 4:** Seroprevalence of anti-dengue virus IgG stratified by age groups and study sites

	West Central	North District	East District	A factory in Ana District	A factory in Yongkang District	Total
Age group	+/N	+/N	+/N	+/N	+/N	+/N
	(%)	(%)	(%)	(%)	(%)	(%)
20–34	6/27	0/23	3/40	11/243	6/177	26/510
	(22.2)	(0.0)	(7.50)	(4.5)	(3.4)	(5.1)
35–49	9/48	8/40	6/65	4/77	9/114	36/344
	(18.8)	(20.0)	(9.23)	(5.2)	(7.9)	(10.5)
50–64	42/101	16/79	22/134	0/5	0/11	80/330
	(41.6)	(20.3)	(16.42)	(0.0)	(0.0)	(24.2)
≥65	35/50	15/33	50/124			100/207
	(70.0)	(45.5)	(40.32)			(48.3)
Total	92/226	39/175	81/363	15/325	15/302	242/1391
	(40.7)	(22.3)	(22.3)	(4.6)	(5.0)	(17.4)
Age-standardized	32.6%	17.7%	14.8%	2.9%	3.5%	17.6%

**Table 5:** Analysis of past dengue virus infection among study population by selected characteristics

		Univariate analysis				Multivariate analysis		
Characteristics	No. positive/No. tested	(%)	Odds ratio	95% CI	*P* value	Adjusted OR	95% CI	*P* value
Gender								
Male	87/586	(14.9)	1.00	Referent	-	1.00	Referent	-
Female	155/805	(19.3)	1.37	1.03,1.82)	0.0327	0.90	(0.64,1.25)	0.5221
Age group (y)					<.0001			<.0001
20–34	26/510	(5.1)	1.00	Referent	-	1.00	Referent	-
35–49	36/344	(10.5)	2.18	(1.29,3.68)	0.0037	1.57	(0.89,2.76)	0.1174
50–64	80/330	(24.2)	5.96	(3.73,9.51)	<.0001	2.98	(1.65,5.37)	0.0003
≥65	100/207	(48.3)	17.40	(10.77,28.10)	<.0001	10.03	(5.42,18.58)	<.0001
Place					<.0001			<.0001
West Central District	92/226	(40.7)	14.19	(7.93,25.40)	<.0001	4.98	(2.49,9.99)	<.0001
North District	39/175	(22.3)	5.93	(3.16,11.11)	<.0001	2.15	(1.02,4.53)	0.0433
East District	81/363	(22.3)	5.94	(3.34,10.54)	<.0001	1.71	(0.84,3.50)	0.1410
Factory in Yongkang	15/302	(5.0)	1.08	(0.52,2.25)	0.8368	1.01	(0.48,2.12)	0.9797
Factory in Annan	15/325	(4.6)	1.00	Referent	-	1.00	Referent	-
Family diagnosed with dengue at any time								
No	205/1289	(15.9)	1.00	Referent	-	1.00	Referent	-
Yes	37/102	(36.3)	3.01	(1.96,4.63)	<.0001	2.07	(1.25,3.43)	0.0045

The elderly subjects (aged ≥65 years) had much higher seroprevalence of anti-DENV IgG compared to other age groups (Table 5). The historical island-wide severe epidemic occurred in 1942–43, we therefore used age ≥ 73 years as the cutoff point since these people likely had exposed to DENV previously. Results revealed that the seroprevalence of anti-DENV IgG among those aged ≥73 years was 71.4%, while only 36.5% among those aged between 65 and 72 years.

### qRT-PCR

There was only one out of 1391 participants (0.07%) positive for DENV RNA by qRT-PCR. This subject was a 38-year old male living in the East District, who did not recall having any symptoms or history of DENV infection at the time of blood drawn. The viral load in collected serum was very high at 3.57 × 10^6^ copies/mL by qRT-PCR and at 1.3 × 10^5^ PFU/mL by standard plaque assays. Viral sequence analysis revealed that the asymptomatic subject was infected with DENV2.

## Discussion

We conducted a serosurvey during the end of the 2015 dengue epidemic in Tainan city situated in southern Taiwan. Although several reviews and guidelines stated that anti-DENV IgM only persisted for 2–3 months [14–16], our recent published findings showed that anti-DENV IgM could persist for about 338.3 days (95% CI 279.7–446.9) assayed by commercial ELISA kits from Standard Diagnostic [17]. Therefore, despite the duration between the beginning of this epidemic and the final recruitment of participants was over 6 months, the positive anti-DENV IgM observed in the current study was a good indicator for being infected in the 2015 epidemic. The overall seroprevalence of anti-DENV IgM and anti-DENV IgG in enrolled subjects was 6.8 and 17.4%, respectively. It was found that districts with high incidence of reported dengue disease in 2015 also had high seroprevalence of anti-DENV IgM. Comparing the overall seroprevalence of anti-DENV IgM (11.5%) with the overall incidence of reported dengue patients (3.1%) in West Central District, North District, and East District, we therefore estimated that 1 out of 3.7 DENV infections was confirmed and reported to health authority in Taiwan in the 2015 epidemic, which was very different from Singapore where it was estimated that 1 out of 23 and 35 of DENV infections were notified in 2004 and 2007, respectively [18, 19]. The unrecognized DENV infection could be attributed to asymptomatic infections or underreporting of symptomatic cases. Previous studies show that the ratio of symptomatic to asymptomatic DENV infection in adults is about 1: 1 to 1: 3 [19–22]. Therefore, our results suggested that most of the symptomatic cases had been reported to the surveillance system in Tainan in 2015. Widespread use of rapid diagnostic tests probably had greatly changed the reporting practice of healthcare professionals. Prior to this epidemic, laboratory confirmation of dengue cases usually took several days in Taiwan due to the samples required to be centralized and sent to the Taiwan Center for Disease Control for testing. Although reporting of suspected dengue cases within 24 h has been mandatory in Taiwan, healthcare professionals were less willing to report in the past as this could falsely trigger outbreak investigation and vector control activities if the patients were later tested negative for DENV infection. Rapid diagnostic tests were extensively used in local clinics and hospitals in the 2015 dengue epidemic in Tainan, which facilitated the diagnosis and reporting of dengue cases. Moreover, the awareness of dengue was escalated among the general public and healthcare workers during this epidemic resulting from numerous dengue campaigns through various media and health educational programs. Therefore, underreporting of dengue fever cases during this epidemic was very low as compared to other countries. Good surveillance system can provide essential information on risk assessment, timely responses, and program evaluation.

The risk of DENV infections in 2015 was found to be increased with age in Taiwan, especially for those aged ≥50 years, which is distinct from many dengue endemic countries where children are most affected [23–25]. In hyperendemic countries, people are likely to have been exposed to all four DENV serotypes in childhood and thus have immunity when they get older, while in non-endemic areas the adults and the elderly are still susceptible to DENV infections. Although this study did not explore detailed risk factors of DENV infection, one previous study showed that larger size of household, piled junks in the basement, and outdoor bonsai were significant risk factors in Taiwan [26]. However, why people in middle age and older had a higher risk of DENV infection in this epidemic is still unknown; further studies to investigate the behavioral and environmental risk factors for these people are required.

One person did not have symptoms and yet had high viremia. We followed up this participant and he did not recall to have any symptoms after blood was drawn. We previously published a mathematical model to estimate the temporal prevalence of asymptomatic dengue viremia in blood donors during the 2015 dengue epidemic in Tainan [11]. This model using a statistical resampling approach with four main parameters including duration of viremia, duration of viremia before symptom onset, apparent-to-inapparent infection ratio, and reporting-to-underreporting ratio. The estimated number of participants with asymptomatic dengue viremia using this model was 1.1, which was calculated by summing up the product of the number of participants recruited in each district on each date and the estimated daily prevalence of asymptomatic dengue viremia of corresponding district and date obtained from the model. We found one people with asymptomatic DENV viremia in our study population, which was very close to what was estimated from the model, supporting the validity of the model. Although dengue is currently not endemic in Taiwan, our model predicts very high prevalences of asymptomatic dengue viremia in hot areas during the peak of dengue epidemic; for example, the estimated maximal daily prevalence of asymptomatic viremia could be as high as 3.3% in West Central District. Therefore, measures to ensure blood safety should be evaluated and implemented, especially in places and time with high estimated prevalence of asymptomatic viremia [27]. In addition to blood safety, a recent study showed that people with inapparent infection could transmit DENV to mosquitoes, despite their lower average level of viremia [28]. People with asymptomatic viremia have higher mobility than those who developed symptoms, and thus may significantly contribute to DENV dissemination in a community or new foci of infection [29]. Indeed, a recent model analysis suggests that inapparent infections may account for 84% of DENV transmission [30].

The overall seroprevalence of anti-DENV IgG remains low in Tainan as compared to other dengue-endemic countries [19, 31–33], especially in the younger age group and in districts with a low incidence of reported cases in 2015. Therefore, explosive dengue outbreaks can still happen in the future due to a large proportion of susceptible people and low level of herd immunity. However, the seroprevalence of anti-DENV IgG among those who were borne during or before the severe island-wide epidemic in 1942–43 was greater than 70%. Since the elderly in Taiwan are more likely to be infected by DENV as shown in our study and had a higher risk of severe disease and death [10, 34], vaccination program targeting this population may be useful to reduce disease burden and mortality. However, the first licensed dengue vaccine CYD-TDC (Dengvaxia®) was recommended for people aged 9–45 years; therefore, studies regarding the efficacy and safety of the vaccine in the senior population is urgently needed [35].

There were several limitations in this study. Firstly, since the study participants were not randomly selected and those who were willing to participant in the study might have different features from those who did not participate, selection bias can be a problem. For example, the surveillance data showed similar incidence of dengue cases in West Central District and North District, but the seroprevalence of anti-DENV IgM in these two districts was very different. Outbreak investigation in the very beginning of the 2015 epidemic revealed that a flea market in the North District was the major source of transmission; therefore, extensive vector control programs were enforced and the market was forced to close subsequently. One of the two recruitment sites in the North District was near the flea market. We found that high-risk people in this area were less willing to participant in our study, probably because they did not want their homes to be labeled as a high-risk area; as a result, the seroprevalence in North District might be underestimated. Secondly, children were not recruited in this study. However, less than 10% of the confirmed cases were aged < 18 years in this epidemic [10]. Thirdly, although commercial anti-DENV IgM ELISA kits have been shown to have very high sensitivity and specificity [36, 37], cross-reactivity with other similar flaviviruses can be a problem for anti-DENV IgG ELISA [38]. Fourthly, every 10 samples were pooled for DENV RNA detection, which would lower the sensitivity and thus the prevalence of asymptomatic viremic people might be underestimated. Finally, detailed information regarding important risk factors for DENV infection were not collected. Due to the unexpectedness of this epidemic, the rapid decline of the epidemic after October in 2015, and the short duration of anti-DENV IgM detection, we had to use an existing study protocol in order to recruit participants in time before anti-DENV IgM waned. However, since our another follow-up study showed that anti-DENV IgM persisted much longer than previously thought using some commercial ELISA kits [17], future researchers who want to conduct a serosurvey after an unexpected dengue epidemic to assess the disease burden can learn from this experience and have more time to design their studies.

## Conclusions

A seroprevalence study of adults was conducted during the ending phase of the 2015 dengue epidemic in Tainan, Taiwan. The overall seroprevalence of anti-DENV IgM and anti-DENV IgG was 6.8 and 17.4%, respectively. Older age, living in areas with a high incidence of reported dengue cases, and having family members being diagnosed with dengue in 2015 were independent risk factors of DENV infection in 2015. The results suggested that the majority of symptomatic cases were reported to the surveillance system, which might be due to the widespread use of rapid dengue diagnostic tests. The results also indicate that the overall herd immunity remains low and the current approved Dengvaxia® is not quite suitable for vaccination in Taiwan.

## Abbreviations

DENV: Dengue virus
ELISA: Enzyme-linked immunosorbent assay
PRNT: Plaque reduction neutralization test
